# Inhibitory effects of cannabidiol on voltage-dependent sodium currents

**DOI:** 10.1074/jbc.RA118.004929

**Published:** 2018-09-14

**Authors:** Mohammad-Reza Ghovanloo, Noah Gregory Shuart, Janette Mezeyova, Richard A. Dean, Peter C. Ruben, Samuel J. Goodchild

**Affiliations:** From the ‡ Department of Biomedical Physiology and Kinesiology, Simon Fraser University, Burnaby, British Columbia V5A 1S6, Canada and; the § Department of Cellular and Molecular Biology, Xenon Pharmaceuticals, Burnaby, British Columbia V5G 4W8, Canada

**Keywords:** sodium channel, cannabinoid, neuron, electrophysiology, central nervous system (CNS), cannabidiol, epilepsy, Kv2.1, phytocannabinoid, voltage-gated sodium channel

## Abstract

*Cannabis sativa* contains many related compounds known as phytocannabinoids. The main psychoactive and nonpsychoactive compounds are Δ9-tetrahydrocannabinol (THC) and cannabidiol (CBD), respectively. Much of the evidence for clinical efficacy of CBD-mediated antiepileptic effects has been from case reports or smaller surveys. The mechanisms for CBD's anticonvulsant effects are unclear and likely involve noncannabinoid receptor pathways. CBD is reported to modulate several ion channels, including sodium channels (Nav). Evaluating the therapeutic mechanisms and safety of CBD demands a richer understanding of its interactions with central nervous system targets. Here, we used voltage-clamp electrophysiology of HEK-293 cells and iPSC neurons to characterize the effects of CBD on Nav channels. Our results show that CBD inhibits hNav1.1–1.7 currents, with an IC_50_ of 1.9–3.8 μm, suggesting that this inhibition could occur at therapeutically relevant concentrations. A steep Hill slope of ∼3 suggested multiple interactions of CBD with Nav channels. CBD exhibited resting-state blockade, became more potent at depolarized potentials, and also slowed recovery from inactivation, supporting the idea that CBD binding preferentially stabilizes inactivated Nav channel states. We also found that CBD inhibits other voltage-dependent currents from diverse channels, including bacterial homomeric Nav channel (NaChBac) and voltage-gated potassium channel subunit Kv2.1. Lastly, the CBD block of Nav was temperature-dependent, with potency increasing at lower temperatures. We conclude that CBD's mode of action likely involves 1) compound partitioning in lipid membranes, which alters membrane fluidity affecting gating, and 2) undetermined direct interactions with sodium and potassium channels, whose combined effects are loss of channel excitability.

## Introduction

The cannabis plant is composed of over 100 compounds known as phytocannabinoids (1). Among these phytocannabinoids, CBD,^2^ is of great interest because of its lack of potency on CB1 and CB2 receptors that are thought to mediate psychotropic activity. Interactions with these receptors by yet another cannabinoid, THC, at submicromolar concentrations cause the well known cannabis effects (2). Recently, reports of the use of CBD as an anticonvulsant agent have been rapidly increasing (3); however, to date, there is no consensus on a well defined mode of action for the CBD-mediated antiepileptic effects.

Because CBD has a lower affinity for the endocannabinoid receptors than THC (4), several studies suggest that the anticonvulsant effects of THC and CBD in maximal electroshock (ED_50_ ∼120 mg/kg, brain concentration = ∼22 μm) and pilocarpine models occur via different mechanisms (5, 6). Whereas the THC activity is mostly on the CB1 receptor, the anticonvulsant effects of CBD are not. These findings have inspired the growth of CB1- and CB2-independent focused research. Many mechanisms have been proposed for the action of CBD on different systems. CBD acts as an agonist on human TRP channels (3–30 μm), and specifically on TRPV1 (7, 8), which is in part responsible for calcium channel modulation (9). CBD also inhibits heterologously expressed Cav3.1, Cav3.2, and native neuronal T-type voltage-gated calcium channels (10). These low-voltage activated channels also can be blocked by other antiepileptic drugs such as zonisamide (11). In addition to Cav channels, CBD inhibits persistent and resurgent sodium currents generated by WT-Nav1.6 channels and the exacerbated versions of these currents in GOF epilepsy-causing Nav1.6-mutants (12). A recent human clinical trial has indicated that CBD (dosed at 20 mg/kg) is efficacious against drug-resistant seizures in Dravet syndrome (3). Previous investigations of the cannabis-mediated blockade of Nav channels have determined that CBD (10 μm) significantly decreases action potential frequency in rat CA1 hippocampal neurons and Nav current density in human neuroblastoma cells and mouse cortical neurons (13). Other studies have used dissociated hippocampal cultures derived from embryonic day 18 rats to measure toxicity and neuroprotective responses to CBD. These studies indicate that the EC_50_ of CBD is within the 1–4 μm range while causing neurotoxicity at 33 μm (14).

The transient sodium current through Nav channels initiates action potentials in neurons, skeletal muscles, and cardiac muscles. Any changes to the gating properties of these channels, and subsequently the current passed through them during an action potential, can cause extremely life-limiting conditions that can sometimes be lethal. Both GOF and LOF in sodium channels disrupt electrical signaling (15–18).

In the primary sodium channel isoforms of the central nervous system, Nav1.1, 1.2, 1.3, and 1.6, both GOF and LOF elicit epilepsy syndromes (15, 19–21). These include relatively mild epilepsies, like benign familial neonatal-infantile seizures, and more severe forms, such as Dravet syndrome (22–24) and early-infantile epileptic encephalopathy-13 (25).

Compounds that inhibit Nav current have been used extensively for clinical treatment of all of the above hyperexcitability disorders (26). In this study, we sought to characterize, in detail, the biophysical effects of CBD on peripheral and central nervous system sodium channel isoforms.

## Results

### CBD is an inhibitor of human sodium currents

**Figure 1.**
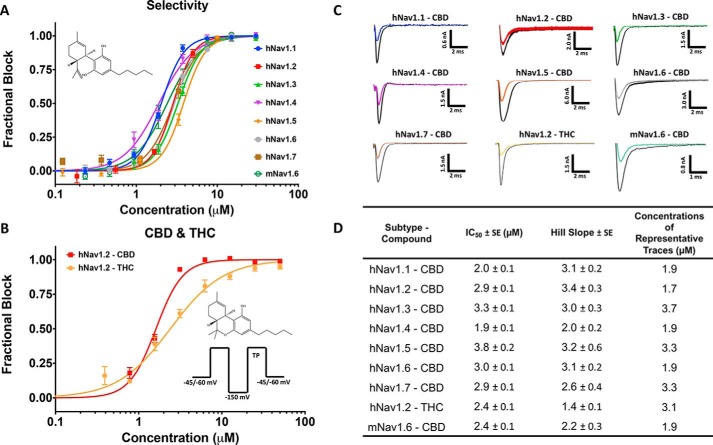
**CBD and THC inhibit Nav currents.** *A*, the IC_50_ curves of CBD block on hNav1.1–1.7 and mNav1.6. The chemical structure of CBD is shown in the *top left corner. B*, THC IC_50_ of hNav1.2 compared with CBD. The pulse protocols used and the chemical structure of THC are shown on the *bottom right*. Channels were exposed to each compound for 20 min. *C*, representative current traces at IC_50_ in each sodium channel. *Traces* are taken from the concentrations that are closest to IC_50_. *D*, table of IC_50_ and Hill slope fitted parameters (*n* = 3–15 cells exposed at each concentration; the S.E. values quoted are errors of the fit).

Previous reports suggested that Nav currents are inhibited by micromolar concentrations of CBD but lacked detailed concentration-response data and defined potencies. We sought to determine whether CBD has any selectivity across the sodium-channel superfamily. Thus, we measured the concentration dependence of inhibition of inactivated-state sodium channels by CBD. Our results demonstrate that CBD inhibits hNav1.1–1.7 with low micromolar potency (Fig. 1, *A* and *D*). To construct the concentration response, individual cells were exposed to single concentrations of CBD; then normalized inhibition at each concentration was pooled and fit with a Hill Langmuir equation, providing IC_50_ and Hill slopes (Fig. 1*A*). Representative current traces at approximately IC_50_ concentrations for each subtype tested are shown in (Fig. 1*C*). Interestingly, the sodium-current inhibition has very steep Hill slopes of >2 across all subtypes (Fig. 1, *A* and *D*). This indicates that CBD does not likely inhibit the channel currents through a 1:1 binding mechanism but instead suggests multiple interactions contribute to this inhibition. Although there is some variation in the IC_50_ values obtained on each subtype, the steep Hill slopes make IC_50_ values extremely sensitive to small changes in concentration, which could account in part for this variation of ∼2-fold in IC_50_. Similar to the hNav channels, CBD also inhibited the mouse Nav1.6 (mNav1.6) current, suggesting that these human IC_50_ values hold in rodent isoforms (Fig. 1, *A* and *D*).

### THC inhibition of Nav currents

The chemical structures of CBD and THC are very similar, with the sole difference being the closure of a ring on THC as opposed to a free hydroxyl group in CBD. Given that this difference is the structural basis for the functional differences between CBD and THC, we tested THC against hNav1.2. Our results suggest that although the potency of the sodium-current inhibition between THC and CBD is similar, the Hill slope associated with THC is less steep (Fig. 1, *B* and *D*). This may indicate that THC has some differences in the mechanism of sodium-current inhibition than CBD.

### CBD prevents Nav channels from opening

**Figure 2.**
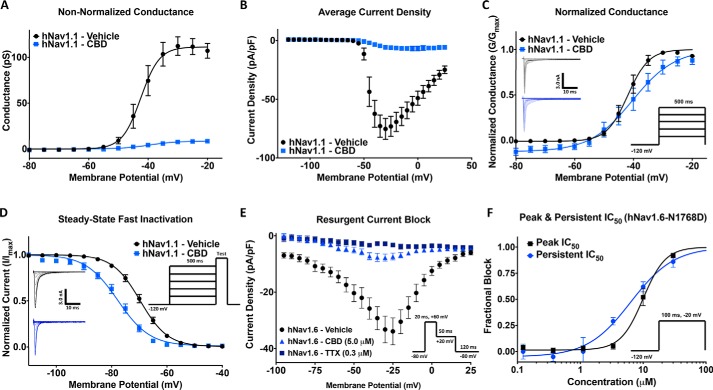
**CBD effects on activation, steady-state fast inactivation (SSFI), resurgent, and persistent currents.** *A*, conductance difference in hNav1. 1 in vehicle and 3.3 μm CBD concentration (vehicle: *V*_½_ = −42.5 ± 0.8, slope = 3.1 ± 0.7, *n* = 11; CBD: *V*_½_ = −39.6 ± 1.8, slope = 4.6 ± 1.5, *n* = 5). *B*, average current density of hNav1.1 in vehicle and CBD (vehicle: current density = −75.3 ± 8.9 pA/pF, *n* = 11; CBD: current density = −6.8 ± 3.0 pA/pF, *n* = 5). *C*, voltage dependence of activation as normalized conductance plotted against membrane potential (vehicle: *V*_½_ = −41.9 ± 0.4 mV, slope = 3.6 ± 0.4, *n* = 7; CBD: *V*_½_ =−40.3 ± 1.0 mV, slope = 7.2 ± 0.9, *n* = 5). *D*, voltage dependence of SSFI as normalized current plotted against membrane potential (vehicle: *V*_½_ = −69.7 ± 0.2 mV, slope = 5.0 ± 0.2, *n* = 12; CBD: *V*_½_ = −77.8 ± 0.3 mV, slope = 5.8 ± 0.3, *n* = 5). *E*, resurgent current block in hNav1.6 (vehicle: resurgent density = −33.9 ± 4.8 pA/pF, *n* = 11; CBD: resurgent density = −7.3 ± 1.2 pA/pF, *n* = 23; tetrodotoxin (*TTX*): resurgent density = −3.3 ± 0.7 pA/pF, *n* = 31). *F*, IC_50_ of CBD block of peak and persistent currents in hNav1.6 mutant (N1768D) (peak: IC_50_ = 10.0 ± 0.7 μm, slope = 2.0 ± 0.4, *n* = 3–11; persistent: IC_50_ = 6.4 ± 1.0 μm, slope = 1.3 ± 0.2, *n* = 3–9).

We next examined the effects of CBD on Nav channel activation by measuring peak channel conductance at membrane potentials between −120 and +30 mV. We show the effects of 3.3 μm CBD on peak sodium-current densities (Fig. 2*A*) and a plot of conductance (Fig. 2*B*). Approximately 90% of the sodium conductance was inhibited. The normalized conductance is plotted against membrane potential, showing that CBD does not induce large changes in either the midpoint (*V*_½_) or apparent valence (slope, *k*) of activation of the available sodium channels (*p* > 0.05) (Fig. 2*C*). Therefore, exposure to CBD at this concentration prevents channels from conducting; however, this exposure does not alter the voltage dependence of activation.

We next measured the voltage dependence of fast inactivation. The normalized current amplitudes at the test pulse is shown as a function of prepulse voltages (Fig. 2*D*). The current at the test pulse was inhibited by more than 90%; however, unlike activation, the voltage dependence of steady-state fast inactivation of the remaining current was hyperpolarized by 8.1 mV (*p* = 0.0002). This indicates CBD increased the propensity for channels to inactivate over the 500-ms prepulse in channels that were not inhibited from opening from rest, suggesting that CBD stabilizes the inactivated state of sodium channels.

It was previously shown that 1 μm CBD inhibits the persistent and resurgent sodium currents in form of epilepsy caused by hNav1.6 GOF mutation, N1768D, which displays a noninactivating component (12). We also found that CBD inhibits the resurgent current induced by including 200 μm β4-peptide to the intracellular solution. Fig. 2*E* shows that 5 μm CBD inhibits the majority of resurgent currents, which is consistent with CBD preventing channels from opening (Fig. 2*A*). Next, we sought to establish the concentration dependence of CBD inhibition of the persistent current of the inactivation-deficient N1768D mutant. We found that persistent currents were inhibited at slightly lower concentrations than the peak currents, suggesting there may be interactions between CBD and the open or inactivated states over the course of a 100-ms depolarization (Fig. 2*F*).

### CBD stabilizes inactivated states of Nav channels

**Figure 3.**
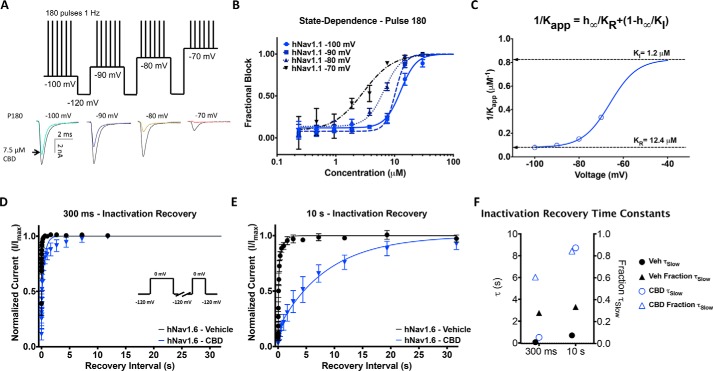
**State-dependent inhibition of Nav current by CBD and effects on recovery from inactivation.** *A*, pulse protocol showing 180 pulses run at 1 Hz at each holding potential and representative current traces. *B*, CBD potency at four holding potentials at pulse 180 (3 min) (IC_50_: −100 mV = 12.7 ± 1.0 μm, −90 mV = 10.3 ± 0.5 μm, −80 mV = 6.7 ± 0.4 μm, −70 mV = 2.9 ± 0.6 μm; *n* = 2–6). *C*, apparent *K_d_* at different voltages was well fit with a four-state model invoking different potencies for resting and inactivated-state block. *D* and *E*, recovery from inactivation in 3.7 μm CBD at: 300 ms (vehicle (*Veh*): τ_Fast_ = 0.00173 s, τ_Slow_ = 0.0688 s, *n* = 35; CBD: τ_Fast_ = 0.00654 s; τ_Slow_ = 0.516 s; *n* = 3) and 10 s (vehicle: τ_Fast_ = 0.0715 s, τ_Slow_ = 0.696 s, *n* = 33; CBD: τ_Fast_ = 0.272 s; τ_Slow_ = 8.72 s; *n* = 3). *F*, the slow components of recovery from inactivation in vehicle and CBD at 300 ms and 3 s are shown on the *left y axis*, and the fraction of slow to fast component of recovery from inactivation is shown on the *right y axis*.

We used a protocol to examine state-dependent inhibition across a range of holding potentials where channel inactivation varies (27). We first held channels at a holding potential of −100 mV where channels are almost all in the resting state, whereas pulsing the channels 180 times at 1 Hz to allow CBD to reach equilibrium. Then we depolarized the holding potential by 10 mV three more times and repeated the pulse train at each voltage (Fig. 3*A*). We show the fractional block of sodium currents from the last pulse (180th) from each holding potential (Fig. 3*B*). Fig. 3*C* shows a plot of the inverse of the apparent IC_50_ fit with a four-state binding model that used parameters obtained from the Boltzmann fit of the voltage dependence of steady-state fast inactivation. This established that the apparent potency is directly related to the proportion of inactivated channels at different holding potentials. Our results demonstrate that CBD inhibits the sodium current from both rest and inactivated states; however, the potency of CBD is ∼10-fold greater for inhibiting inactivated compared with resting states (Fig. 3*C*).

To assess the time dependence and degree of stabilization of the inactivated state, we then measured the recovery from inactivation of hNav1.6 in the presence of 3.7 μm CBD (Fig. 1*A*). This was done after depolarizing prepulse durations of 300 ms and 10 s, from a holding potential of −120 mV. These prepulse durations correspond to inactivation recovery from fast and slow inactivated states, respectively. The mean normalized recovery following the prepulse in CBD and control conditions were plotted and fit with a biexponential function (Fig. 3, *D* and *E*). The τ_Slow_ and fraction of the recovery fit with τ_Slow_ are plotted in Fig. 3*F*, which shows that CBD increases the fraction of recovery that is slow and the time constant of the slow component of recovery from inactivation from 300 ms to 10 s. This indicates that CBD slows the recovery from inactivation, supporting the hypothesis that CBD stabilizes the inactivated states (Fig. 3, *D–F*).

### CBD potency is increased at lower temperatures

**Figure 4.**
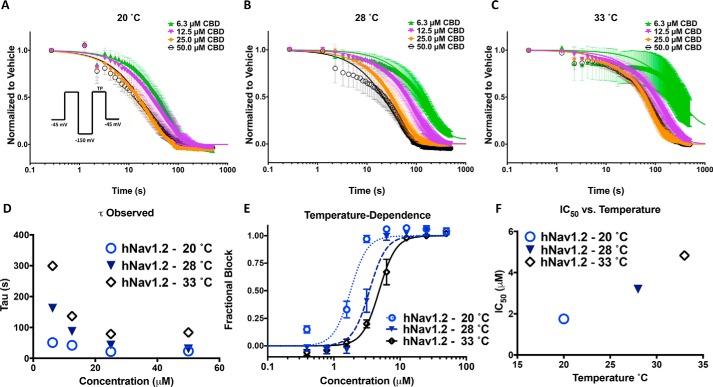
**Lower temperatures increase kinetics and potency of CBD inhibitory effects.** *A–C*, the kinetics of CBD block at 20, 28, and 33 °C at 50. 0, 25.0, 12.5, and 6.3 μm (20 °C: 6.3 μm = 51.0 ± 0.6 s, 12.5 μm = 42.2 ± 0.3 s, 25.0 μm = 21.6 ± 0.4 s, 50.0 μm = 23.3 ± 0.3 s; 28 °C: 6.3 μm = 162.6 ± 0.9 s, 12.5 μm = 87.7 ± 0.2 s, 25.0 μm = 42.8 ± 0.1 s, 50.0 μm = 30.4 ± 0.4 s; 33 °C: 6.3 μm = 299.0 ± 9.5 s, 12.5 μm = 137.0 ± 0.5 s, 25.0 μm = 79.2 ± 0.3 s, 50.0 μm = 84.1 ± 0.3 s, *n* = 10–14). The variability at the lower concentration of 6.3 μm at 33 °C is larger because of the slowing of CBD effect. *D*, time constants associated with the plots at *A–C. E*, IC_50_ at the noted temperatures (IC_50_ (μm): 20 °C = 2.1 ± 0.1, 28 °C = 3.4 ± 0.1, 33 °C = 4.7 ± 0.2, *n* = 2–11), the slope factor is fixed at 3.4. *F*, relationship of CBD potency as a function of temperature.

Binding kinetics are typically responsive to changes in temperature, with higher temperatures increasing the rates of compound equilibration. To further investigate the mode of CBD interaction with sodium channels, we measured the observed rates of equilibration (time constant observed, τ_obs_) of inhibition by fitting single exponential decays to inhibition of currents at three different temperatures. We examined the kinetics at concentrations above the IC_50_ to ensure a clear inhibition signal window to define the τ_obs_ (Fig. 4, *A–C*). Channels were held at −45 mV and pulsed at 1 Hz following a recovery pulse to −150 mV for 60 ms, and CBD was rapidly applied to the cells at different concentrations (Fig. 4*A*). The fraction of inhibition was normalized against the response in vehicle and plotted against the time elapsed after CBD addition, which was set at *t* = 0. The inhibition was then fit with a single exponential function to obtain τ and plotted against concentration. We show that τ_obs_ saturated at a minimum with increasing concentrations, counter to the prediction of a single two-state ligand-binding reaction, which predicts a continually increasing τ_obs_ with increasing compound concentrations (Fig. 4*D*). This suggests a rate-limiting step in the inhibitory pathway that is not dependent on the concentration of CBD in bulk solution as would be expected for binding to a specific inhibitory site on the channel. Interestingly, the kinetics of CBD inhibition were also found to be more rapid at cooler temperatures (Fig. 4*D*). This is the opposite of what is expected for a classic inhibitor in a two-state binding model, where the binding and unbinding rate constants, *k*_on_ and *k*_off_, are intrinsically temperature-dependent. These results further suggest that CBD does not inhibit conductance through a direct interaction with a single specific binding site on the channel. We also found that the potency of CBD is increased at the lower temperatures (Fig. 4, *E* and *F*).

### CBD does not inhibit via interactions at the classic local-anesthetic pore-binding site

**Figure 5.**
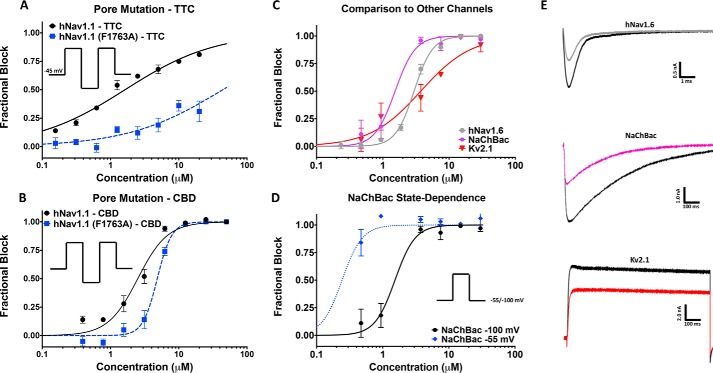
**CBD block of F1763A mutant, NaChBac, and Kv2.1.** *A*, TTC block of hNav1.1 (hNav1.1, IC_50_ = 1.6 ± 0.1 μm, slope = 0.7 ± 0.03; Phe^1763^, IC_50_ ∼ 44.1 μm) with and without the F1763A mutation. *B*, F1763A causes a slight decrease in CBD potency in hNav1.1 (hNav1.1: IC_50_ = 2.5 ± 0.2 μm, slope = 2.0 ± 0.2, *n* = 2–6; F1763A: IC_50_ = 4.8 ± 0.2 μm, slope = 4.1 ± 0.6, *n* = 3–811 cells exposed at each concentration). *C*, shows a comparison of the CBD inhibition of hNav1.6, NaChBac, and Kv2.1 (hNav1.6: IC_50_ = 3.0 ± 0.1 μm, slope = 3.1 ± 0.2, *n* = 2–6; NaChBac: IC_50_ = 1.5 ± 0.2 μm, slope = 2.8 ± 0.9, *n* = 1–4; Kv2.1: IC_50_ = 3.7 ± 0.8 μm, slope = 1.1 ± 0.2, *n* = 1–5 11 cells exposed at each concentration). *D*, shows the state-dependent block of NaChBac tested at −55 and −100 mV (−100 mV: IC_50_ = 1.5 ± 0.2 μm, slope = 2.8 ± 0.9, *n* = 1–4; −55 mV: IC_50_ = 0.24 ± 0.05 μm, slope = 2.8 ± 0.9, *n* = 1–311 cells exposed at each concentration). *E*, current traces associated with the channels shown in *C*.

Because CBD inhibition shares a characteristic of classic pore blockers (preference for the inactivated state), we created a pore mutation in the local-anesthetic receptor site in hNav1.1 (F1763A) to determine whether the CBD potency was affected. The Phe^1763^ residue is part of a well established binding site for many of the most common local anesthetics, including tetracaine (TTC) (28). To avoid any impacts on potency being caused by the shifts in stability of inactivation in this mutant (WT-hNav1.1 inactivation *V*_½_ = −62.0 ± 0.4 mV, slope = 7.2 ± 0.4, *n* = 3; F1763A inactivation *V*_½_ = −49.3 ± 0.1 mV, slope = 6.4 ± 0.1, *n* = 17), we measured inhibition from a holding potential of −45 mV where both channels were >50% inactivated. To validate the F1763A-mutant channels, we also measured the potency of TTC and compared the results against WT-hNav1.1, which showed a drop in potency (Fig. 5*A*). For CBD, the F1763A-mutant channels only caused a slight drop in potency of ∼2-fold (Fig. 5*B*). Molecular docking using a homology model of hNav1.1 based on the eukaryotic cockroach cryo-EM structure (Navpas) (Fig. S1*A*) suggested that CBD may in fact bind close to Phe^1763^. This suggests that Phe^1763^ is not a primary determinant of CBD inhibition but does not rule out the possibility that CBD may interact with other pore residues.

To determine whether CBD could also inhibit other nonhuman Nav channels, we tested it on the bacterial homomeric Nav channel (NaChBac) (Fig. 5*C*). Interestingly, we found that NaChBac is also blocked by CBD, although with a slightly greater potency (Fig. 5*C*). Unlike mammalian Nav channels, NaChBac does not inactivate quickly (29). We hypothesized that if the previously observed state dependence of CBD block in hNav channels depends upon the fast-inactivation process, then NaChBac should not show such a state dependence. The potency measured at −100 and −55 mV suggests that state dependence also exists in NaChBac; moreover, this effect may be even more pronounced than in hNav channels (Fig. 5*D*). This may implicate that CBD is not dependent upon, or selective among, different modes of Nav inactivation.

Our findings in the homotetrameric NaChBac raise the question of whether CBD might also inhibit voltage-gated potassium channels, which are also homotetrameric. Our results indicate that CBD also inhibits the Kv2.1 current. Current traces are shown (Fig. 5*E*). These findings, along with the previous reports of CBD modulation of calcium-channel currents, support the idea that CBD is a polypharmacological inhibitor of voltage-dependent ionic currents (10).

### CBD does not alter trafficking

Because CBD nonspecifically inhibits voltage-dependent currents, we sought to establish whether incubation of CBD could affect the channel trafficking to the cell surface over the time scale of our voltage-clamp experiments. To address this question, we assessed membrane-channel distribution in cells incubated overnight with CBD. The results indicate that overnight incubation in 5 μm CBD does not alter sodium channel trafficking and therefore would not affect trafficking on time scales of the voltage-clamp experiments (<20 min of exposure) (Fig. S1, *B–D*).

### CBD inhibits Nav currents in h-iPSC neurons

**Figure 6.**
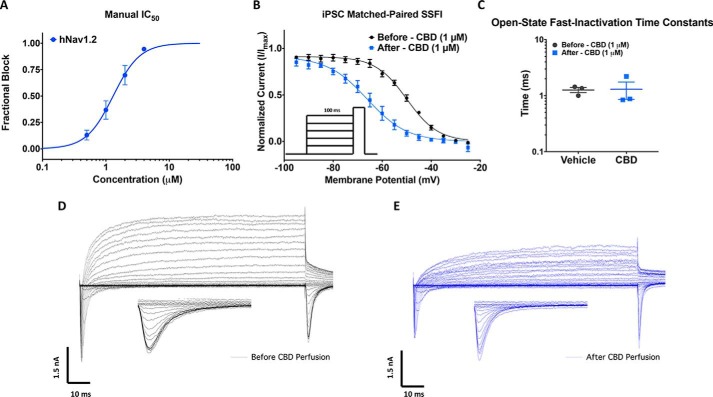
**CBD inhibits human iPSC neuronal Nav and Kv currents.** *A*, concentration-response relationship for CBD inhibition of hNav1.2 channels obtained using manual patch clamp (IC_50_ = 1.3 ± 0.1 μm, slope = 2.1 ± 0.4, *n* = 3–6). *B*, plot of normalized SSFI after a 100 ms prepulse before (*V*_½_ = −50.0 ± 0.4 mV, slope = 6.4 ± 0.4, *n* = 3) and after (*V*_½_ = −66.1 ± 0.7 mV, slope = 8.1 ± 0.6, *n* = 3) perfusion. *C*, open-state fast-inactivation time constants shown on log scale on the *y* axis at −20 mV for vehicles (−20 mV: 1.3 ± 0.1 ms, *n* = 3) and CBD (−20 mV: 1.3 ± 0.4 ms, *n* = 3) in iPSC neurons. *D* and *E*, representative current-voltage relationship recorded from iPSC neurons with a KF based internal solution before 1 μm perfusion of CBD (*D*) and after perfusion (*E*). *Insets* in the panel are zoomed-in view of currents at the test pulse used to assess availability.

To determine whether our observations in HEK cells translate to native neuronal voltage-dependent currents, we measured the effects of CBD on human iPSC neurons. First, we established that potency established with manual patch-clamp methodology, using continuous perfusion of compound, correlated with Qube data by measuring the potency of sodium-current inhibition of HEK cells expressing hNav1.2. Fig. 6*A* shows the mean normalized concentration response plot, which gave an IC_50_ value similar to the Qube value for hNav1.2. The slightly increased potency in the manual assay can be explained by the temperature dependence data (Fig. 4), because manual voltage clamp was performed at ∼20 °C (room temperature), and the Qube IC_50_ values were established at 28 °C (Fig. 1*A*). Consistent with our previous results in HEK cells, both the neuronal sodium and potassium currents were blocked ∼50% by 1 μm CBD as shown by the representative families of current traces in Fig. 6 (*D* and *E*). CBD also caused a hyperpolarization of ∼16 mV in the steady-state inactivation in the remaining available Nav channels in iPSCs (*p* = 0.0031), which was similar to the shifts observed in hNav1.1 in HEK cells (Figs. 2*D* and 6*B*). We also measured the rate time constant of open-state fast inactivation at −20 mV, which did not differ significantly before and after CBD perfusion (*p* > 0.05) (Fig. 6*C*).

### CBD reduces neuronal excitability in a Hodgkin–Huxley model of cortical neuron

**Figure 7.**
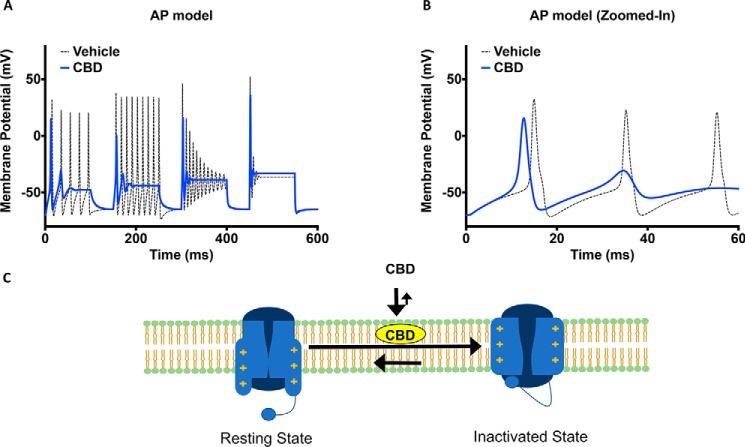
**CBD reduces excitability in an action-potential model and schematic representation of CBD's mode of action.** *A*, simulation of the effects of CBD on action potential morphology over a series of increasing current injection intensities. *B*, zoomed-in simulation of action potentials from the first interval shown in *A. C*, proposed mode of action of CBD involving interactions with both the membrane, resting and inactivated sodium channels.

To test the effect of IC_50_ concentrations of CBD on neuronal excitability, we used a modified version of the Hodgkin–Huxley model to simulate a cortical neuron's excitability (30, 31). In the CBD condition, the sodium and potassium conductances were reduced by 50%. The activation and inactivation *V*_½_ and slopes were taken from the results shown in (Fig. 2, *C* and *D*). In our simulations, the channels were given a series of stepwise current injections with increasing intensities at each step for 100 ms. Each 100-ms step was followed by a 50-ms recovery period in which no current injection was applied (Fig. 7*A*). Our results suggest that the peak amplitude of the first action potential in CBD is smaller than vehicle. This is consistent with the reduction of peak sodium conductance caused by CBD. Furthermore, in the CBD condition we observed that threshold for the first action potential after depolarization was reduced (Fig. 7*B*). The overall effect of CBD on the action potential morphology is that at all current injection intensities, CBD reduces the number of action potentials, leading to a net loss of excitability. These results are consistent with previous studies using current clamp recordings and may in part theoretically explain the reported efficacy of CBD in treating conditions including epilepsy and pain (32).

## Discussion

CBD has a weak affinity for CB receptors; thus, its anticonvulsant properties are attributed to modulations of other targets, including ion channels. Our study suggests that the Nav channel family is among the possible ion channel targets of CBD.

Our results in this study suggest that CBD as a sodium-channel inhibitor is nonselective; therefore, we performed some of our experiments on single sodium-channel subtypes, including the temperature dependence and channel-trafficking assays. We believe that because of the nonspecific nature of the interactions, the results obtained from single subtypes will be generalizable to the whole sodium-channel superfamily. This idea is supported by the consistencies we observed between HEK cells that express only a single sodium-channel subtype, and iPSC neurons, which host several sodium-channel subtypes in a native environment.

### Mechanisms of sodium-current inhibition

The initial results obtained from the selectivity experiments on hNav channels suggested that CBD is indeed a sodium-current inhibitor. However, this inhibition is relatively nonselective in nature and has a steep average Hill slope, suggesting multiple interactions. Like CBD, we found that THC also inhibits hNav1.2, albeit with a less steep Hill slope. Our results indicated that CBD prevents the activation of sodium channels from rest while also stabilizing the inactivated states of these channels without altering the voltage dependence of activation. This inhibition has similarities to that reported for amphiphilic compounds (33, 34). It is suggested that amphiphilic compounds tend to localize at the solution–bilayer interface. This occurrence is made possible by having the polar group of the amphiphile residing at the interface with the hydrophobic region, which then gets inserted into the core of the bilayer. This partitioning into the lipid bilayer alters the biophysical properties of the membrane by reducing stiffness, changing phase preference and curvature. The net effect of these alterations to the membrane is an increased preference for the bilayer-embedded Nav channel in its inactivated state (34).

The similarity of CBD to amphiphiles, along with the multimodal interaction relationship between this compound and the voltage-dependent sodium currents suggested three potential mechanisms culminating in inhibition: 1) multiple direct interactions between CBD and Nav channels, 2) CBD alteration of membrane biophysical properties, or 3) a combination of direct interactions with Nav channels and changes in the membrane fluidity.

### Evidence for CBD effects on sodium channels through altering membrane fluidity

Contrary to what is expected for classic pseudo–second order bimolecular blocking scheme, CBD inhibition curves displayed a Hill slope of ∼3, and compound was fastest to equilibrate and most potent at lower temperatures, indicating that CBD does not follow a classic blocking scheme (Fig. 4). One potential explanation for the decreased potency at 33 °C is that higher temperatures increase the thermal fluctuations of the sodium channels, which become faster, and consequently, it becomes harder for CBD to find interaction sites that may reside in thermally volatile regions of the structure. A second potential explanation is that because the fluidity of the membrane changes as a function of temperature (35), higher CBD concentrations are required to have the same reduction of stiffness at 33 °C where the membrane is more fluid than at lower temperatures, and the relative reduction in stiffness is more pronounced.

### Evidence for direct binding of CBD to the channels to cause inhibition

The observation of an inactivated-state preference, which is a characteristic of Nav pore blocking local anesthetics, led us to investigate the CBD block of a hNav1.1 mutant (F1763A) with a mutation in the local-anesthetic pore-binding site. We found that CBD's inhibitory effects were minimally affected in the F1763A mutants. This suggests that binding at this site is not via similar interactions to local anesthetics (28, 36), although molecular docking supports the possibility that CBD could interact in the pore of hNav1.1.

We found that CBD also inhibits a nonmammalian sodium channel, NaChBac, and the voltage-gated potassium channel, Kv2.1. Interestingly, the CBD block of NaChBac was slightly more potent than the mammalian orthologs. This increased potency might be due to the presence of 4-fold symmetry in these channels, and thus, the four-time repeating of a domain that may be involved in inhibition. Indeed, the equivalent Phe residue in the pore of homotetrameric NaChBac that contributes to local-anesthetic binding (37) (Phe^200^) is repeated four times, as opposed to the single Phe^1763^ in the monomeric hNav1.1, where the F1763A mutation in Nav1.1 induced a small drop in potency. These results offer some support to the hypothesis that, in addition to altering membrane properties, CBD might also have direct interactions with Nav channels that alter conductance. Additionally, our observation that membrane Nav channel distribution was unchanged in cells after a 24-h incubation with CBD suggested there were no effects on trafficking.

### CBD inhibits heterologously expressed and native hNav currents

To assess whether the observed CBD inhibitory effects on voltage-dependent currents in HEK cells could be replicated in the native neuronal environment, we assessed inhibition of currents in iPSC neurons and saw significant inhibition of both Nav and Kv currents (Fig. 5). The potency was comparable with the effects seen in HEK cells stably expressing Nav channels, indicating the HEK cell is a valid expression system to characterize Nav inhibition by CBD.

### Mechanism of sodium-current inhibition by CBD

We found CBD to be highly lipid bound in a TRANSIL brain–lipid binding assay (Table 1). This supported the possibility that the observed effects might be due to alterations to the biophysical properties of the membrane. However, consistent with previous studies (38), we found that phenytoin, a well established nonselective Nav blocker also had high lipid binding (98.5%; Table 1), and it is assumed that phenytoin's efficacy against seizures is via direct Nav channel pore block (39–41). The reported brain exposure levels required for efficacy in rodent maximal electroshock seizure models were also comparable between CBD and phenytoin, supporting the possibility that CBD could at least in part mediate its efficacy through sodium-channel inhibition (Table 1) (6, 39, 41–46). This similarity in lipid binding, however, raises the question of why the two compounds inhibit through different mechanisms. When comparing other physicochemical properties between the compounds, it is striking that the calculated LogD of CBD and phenytoin are 6.6 and −0.7, respectively (ChEMBL accession numbers CHEMBL190461 and CHEMBL16). In contrast to TRANSIL, which measures lipid binding, the LogD is derived by measuring the partitioning of small molecules between octanol and water at pH 7.4 and depends on a variety of structural interactions, including hydrogen bonds and van der Waals interactions. However, because octanol is structurally different from membrane phospholipid bilayers, which contain both polar and nonpolar moieties, it cannot model these interactions well. Consequently, it is possible for compounds to have similar TRANSIL (lipid binding) numbers and different LogD, as found for these compounds. This may suggest that despite having similar lipid-binding percentages (Table 1), the much lower LogD of phenytoin underlies its action as a classic sodium-channel blocker that can enter the pore. In contrast, CBD's very high LogD could result in its accumulation deeper inside the bilayer hydrophobic regions that leads to a greater effective change in the membrane stiffness than phenytoin and underlies CBD's different mode of current inhibition compared with phenytoin. This disconnect between LogD and lipid-binding fractions is further supported by considering another antiepileptic sodium-channel pore blocker, carbamazepine, that has a lower lipid-binding percentage (83.7%) than phenytoin, while having a higher LogD (2.0) than phenytoin (Table 1) (ChEMBL accession number CHEMBL41543). We therefore suggest that high lipid binding alone is not a sufficient property for a compound to alter membrane stiffness, which results from a combination of lipid binding with other physicochemical properties unique to CBD over phenytoin and carbamazepine. Overall, our results suggest that CBD has a nonselective inhibitory effect on voltage-dependent sodium currents. The results presented in this study suggest that this mechanism likely involves a combination of direct interactions with channel hydrophobic regions, possibly the pore, and changing the membrane bilayer flexibility through lipid accumulation, as illustrated by the cartoon in Fig. 7*C*.

**Table 1:** **The comparison of CBD to phenytoin and carbamazepine**

Parameter	Cannabidiol	Phenytoin	Carbamazepine
Brain–lipid binding–TRANSIL (%)	99.6	98.5	83.7
Plasma–protein binding (%)	∼95–99*^a^*	95*^a^*	∼70*^a^*
IC_50_ on Nav channels (μm)	1–4	∼10*^a^*	∼25*^a^*
ED_50_ MES (mg/kg)	∼120*^a^*	8.2–17.5*^a^*	7.5*^a^*
Efficacious plasma level (μm)	6.4–8.3*^a^*	4–10*^a^*	5–12*^a^*
Brain concentration (μm)	21.9*^a^*	86*^a^*	9.7*^a^*
Brain/IC_50_ (ratio)	5.5–21.9	8.6	0.4

*^a^* These parameters were taken from literature (5, 6, 39, 41–48, 57–59).

Results from previous studies along with our findings in this study lead us to conclude that the underlying mechanism for the reported efficacy of CBD against seizures likely involves many systems beyond just CB receptors (7), G protein–coupled receptors (50), or ion channels (10, 12). Dravet syndrome is primarily caused by a heterozygous LOF of Nav1.1 in the brain (51). As such, clinically used sodium-channel blockers are often considered proconvulsant in Dravet syndrome (52, 53), presumably because they inhibit the reduced Nav1.1 current even further. This raises the question of why CBD, as a nonselective Nav inhibitor, would act as an anticonvulsant in Dravet syndrome. A recent study, however, unexpectedly demonstrated efficacy with a nonselective sodium-channel blocker in a Dravet mouse model, suggesting that Nav inhibition can be protective (54). In addition, another study in a Dravet mouse model suggested that the efficacy of CBD against Dravet syndrome may be mediated through antagonism at GPR55 receptors independently of Nav channels (50).

The promiscuity of CBD interactions that have been reported with multiple targets suggests that this compound, like many natural products, is a pleiotropic compound with a complex polypharmacology. It is therefore unlikely that any efficacy against epilepsy is related solely to inhibitory effects on Nav channels; however, our study suggests that inhibition of Nav currents could occur at therapeutically relevant concentrations and thus might contribute to efficacy against seizures.

## Materials and methods

### Cell culture

Suspension HEK-293 cells were used for automated patch-clamp experiments. HEK-293 cells were either stably transfected using the inducible protein-expression T-REx^TM^ system or transiently transfected using Lipofectamine 2000 with sodium α-subunit cDNA constructs. The human *SCN1B* cDNA construct was co-transfected into each cell line. The stable cell lines were maintained under Zeocin (5 μg/ml) and G418 (1 mg/ml) selection in Dulbecco's modified Eagle's medium, 10% fetal bovine serum, and 2 mm l-glutamine medium. The hNav1.1 F1763A and the hNav1.6 N1768D mutations were generated using the QuikChange site-directed mutagenesis kit (Agilent Technologies). GABAergic iPSC neurons were purchased from Cellular Dynamics International (Madison, WI). The cells were seeded at 20–40% confluency onto glass coverslips and cultured up to 4 weeks according to user's guide provided by the vendor. All cells were incubated at 37 °C/5% CO_2_. All cell culture reagents were purchased from Thermo Fisher Scientific (Waltham, MA) unless otherwise noted.

### Automated patch-clamp

Automated patch-clamp recording was used for all experiments unless otherwise noted. HEK cell lines stably expressing the full-length cDNAs coding each Nav α-subunit were generated. The human β1-subunit was co-expressed in each of internally generated cell lines. GenBank^TM^ accession numbers for α-subunits were: human Nav1.1, NM_006920; hNav1.2, NM_021007; hNav1.3, AF225987; hNav1.4, NM_000334; hNav1.5, AC137587, *SCN5A*; hNav1.6, NM_014191; mNav1.6, NM_001077499; and hNav1.7, NM_002977. Sodium currents were measured in the whole-cell configuration using a Qube-384 (Sophion A/S, Copenhagen, Denmark) automated voltage-clamp system. To determine inhibition of the inactivated state, the membrane potential was maintained at a holding potential in which inactivation of the channel subtype is complete. The membrane holding voltages used for subtype selectivity experiments were −60 mV for hNav1.5 and hNav1.7 and −45 mV for hNav1.1, hNav1.2, hNav1.3, hNav1.4, hNav1.6, and mNav1.6. In the protocol used to assess inhibition for hNav1.5 and hNav1.7, a brief hyperpolarization to a negative (*V*_hold_ = −150 mV) voltage for 20 ms was applied at 1 Hz to recover fast-inactivated channels but not compound bound channels, and then a 20-ms test pulse to 0 mV was applied to quantify the fractional availability of channels. For hNav1.1, hNav1.2, hNav1.3, hNav1.4, and hNav1.6, the recovery period was 60 ms applied at a frequency of 0.05 Hz. Intracellular solution contained 120 mm CsF, 10 mm NaCl, 2 mm MgCl_2_, 10 mm HEPES, adjusted to pH 7.2 with CsOH. The extracellular recording solution contained 145 mm NaCl, 3 mm KCl, 1 mm MgCl_2_, 1.5 mm CaCl_2_, 10 mm HEPES, adjusted to pH 7.4 with NaOH. For some studies, a flipped Na^+^ gradient was used where the extracellular solution contained 125 mm choline chloride, 1 mm NaCl, 5 mm KCl, 2 mm CaCl_2_, 1 mm MgCl_2_, 10 mm HEPES/NaOH, pH 7.4, and the intracellular solution contained 115 mm NaF, 15 mm CsCl_2_, 5 mm CsF, 3 mm Na2ATP, 0.3 mm Na2GTP, 2 mm MgCl_2_, 0.1 mm CaCl_2_, 10 mm EGTA, and 10 mm HEPES/CsOH, pH 7.2. Liquid junction potentials calculated to be ∼7 mV were not adjusted for. The currents were low-pass-filtered at 5 kHz and recorded at 25 kHz sampling frequency. Series resistance compensation was applied at 100%, and leak subtraction was enabled. The Qube-384 temperature controller was used to manipulate recording chamber temperature for certain experiments. The rest of the measurements were obtained at room temperature, which corresponds to 27 ± 2 °C at the recording chamber. Appropriate filters for cell membrane resistance (typically >500 MOhm), series resistance (<10 MOhm), and Nav current magnitude (>500 pA at a test pulse from a resting holding potential of −120 mV) were routinely applied to exclude poor quality cells. Vehicle (0.5% DMSO) controls were run on each plate to enable correction for any compound-independent decrease of currents over time. Baselines were established after 20 min in vehicle. Fractional inhibition was measured as current amplitude from baseline to maximal inhibition after 20 min of exposure to test compound unless otherwise noted. Maximal inhibition was established by application of 300 nm tetrodotoxin to each well at the end of the experiment apart from for Nav1.5, for which 30 μm tetracaine was used. Normalized mean inhibition data were fit to the Hill–Langmuir equation,

(Eq. 1) $$Y={\left[\text{C}\right]}^{\text{h}}/\left({\text{IC}}_{50}^{\text{h}}+{\left[\text{C}\right]}^{\text{h}}\right)$$

to estimate the half-maximal inhibitory concentration (IC_50_ value), where *Y* is the normalized inhibition, *C* is the compound concentration, IC_50_ is the concentration of test compound to inhibit the currents 50%, and *h* is the Hill coefficient. Data analysis was performed using Analyzer (Sophion A/S, Copenhagen, Denmark) and Prism (GraphPad Software Inc., La Jolla, CA) software. The IC_50_ values on the Qube-384 automated voltage-clamp platform were generated from Hill equation fits to pooled data at each concentration and so do not have standard errors.

### Manual patch-clamp

Whole-cell patch-clamp recordings were obtained using an Axopatch 200B patch-clamp amplifier (Molecular Devices, Sunnyvale, CA) controlled and recorded using pClamp8 (Molecular Devices). The recording pipette intracellular solution contained 120 mm CsF, 10 mm NaCl, 2 mm MgCl_2_, 10 mm HEPES, adjusted to pH 7.2 with CsOH. Pipette resistances were 2–5 MOhm. The extracellular recording solution contained 145 mm NaCl, 3 mm KCl, 1 mm MgCl_2_, 1.5 mm CaCl_2_, 10 mm HEPES, adjusted to pH 7.4 with NaOH. Currents were low-pass-filtered at 5 kHz and recorded at 25 kHz sampling frequency using a Digidata1440 (Molecular Devices). Series resistance compensation was applied at 60–80%. The experiments were performed at room temperature 19–20 °C. Liquid junction potentials calculated to be ∼7 mV were not adjusted for.

### Compound preparation

CBD was purchased from Cayman Chemicals, and THC was purchased from Toronto Research Chemicals in powder form. Powdered CBD and THC were dissolved in 100% DMSO to create stock. The stock was used to prepare drug solutions in extracellular solutions at various concentrations with no more than 0.5% total DMSO content.

### Manual patch-clamp IC_50_ measurements

IC_50_ measurements were made while holding the membrane voltage at −45 mV. To activate sodium channels, a 60-ms prepulse to −150 mV was used to recover channels from fast inactivation, followed by a 10-ms pulse to −20 mV to open the channel. Using this protocol at a 1-Hz pulse rate, the membrane voltage is maintained at −45 mV 97% of the time. The peak of the inward sodium current at −20 mV was measured, and a stable baseline was established prior to perfusion with compound, after which increasing concentrations of compound were perfused, and the resulting currents were measured after equilibrium was obtained.

### Activation protocols

To determine the voltage dependence of activation, we measured the peak current amplitude at test pulse potentials ranging from −100 mV to +80 mV in increments of +5 mV for 100 ms. Channel conductance (*G*) was calculated from peak *I*_Na_,

(Eq. 2) $$G_{\text{Na}}=I_{\text{Na}}/V-E_{\text{Na}}$$

where *G*_Na_ is conductance, *I*_Na_ is the peak sodium current in response to the command potential *V*, and *E*_Na_ is the Nernst equilibrium potential. The calculated values for conductance were fit with the Boltzmann equation,

(Eq. 3) $$G/G_{\max}=1/\left(1+\exp\left[V_{1/2}-V_{\text{m}}\right]/k\right)$$

where *G*/*G*_max_ is normalized conductance amplitude, *V*_m_ is the command potential, *V*_½_ is the midpoint voltage, and *k* is the slope.

### Steady-state fast-inactivation protocols

The voltage dependence of fast inactivation was measured by preconditioning the channels from −120 to +10 mV in increments of 5 mV for 100 ms, followed by a 10-ms test pulse during which the voltage was stepped to −20 mV. Normalized current amplitudes from the test pulse were fit as a function of voltage using the Boltzmann equation,

(Eq. 4) $$I/I_{\max}=1/\left(1+\exp\left[V_{1/2}-V_{\text{m}}\right]/k\right)$$

where *I*_max_ is the maximum test pulse current amplitude.

### State dependence protocols

To determine state dependence, potency was measured from four different holding potentials (−100, −90, −80, and −70 mV). The protocol started with a holding potential of −100 mV followed by 180 × 20-ms depolarizing pulses to 0 mV at 1 Hz. Then the holding potential was depolarized by 10 mV, and the 180-pulse protocol was repeated until −70 mV was reached.

### Recovery from inactivation protocols

Recovery from inactivation was measured by holding the channels at −120 mV, followed by a depolarizing pulse to 0 mV, and then the potential was returned to −120 mV. This was followed by a depolarizing 10-ms test pulse to 0 mV to measure availability. Recovery from inactivation was measured after prepulse durations of 300 ms and 10 s and fit with a biexponential function of the form,

(Eq. 5) $${\text{Span}}_{\text{Fast}}=\left(Y0-\text{Plateau}\right)*{\text{Percent}}_{\text{Fast}}*0.01$$

(Eq. 6) $${\text{Span}}_{\text{Slow}}=\left(Y0-\text{Plateau}\right)*\left(100-{\text{Percent}}_{\text{Fast}}\right)*0.01$$

(Eq. 7) $$Y=\text{Plateau}+{\text{Span}}_{\text{Fast}}*\exp\left(-K_{\text{Fast}}*t\right)+{\text{Span}}_{\text{Slow}}*\exp\left(-K_{\text{Slow}}*t\right)$$

where *t* is time in seconds, *Y*_0_ is the *y* intercept at *t* = 0, *K*_Fast_ and *K*_Slow_ are rate constants in units the reciprocal of *t*, and Percent_Fast_ is the fraction of the *Y* signal attributed to the fast decaying component of the fit.

### Kinetics at varying temperatures

The kinetics of CBD block were measured at three temperatures: 20, 28, and 33 °C. The channels were held at −45 mV followed by a recovery pulse to −150 mV for 60 ms. The blocked sodium current was normalized to vehicle and subsequently fit with a single exponential function.

(Eq. 8) Y=(Y0−Plateau)∗exp⁡(−K∗t)+Plateau

### Action-potential modeling

Neuronal action-potential modeling was based on a modified Hodgkin–Huxley model (30). The model was modified to match the properties of cortical pyramidal cells (31). The modified parameters were based on electrophysiological results obtained from whole-cell patch-clamp experiments in this study. The model accounted for activation voltage dependence, steady-state fast-inactivation voltage dependence, and peak sodium currents.

### Homology modeling

Homology modeling was performed using the Swiss-Model server. The cryo-EM sodium channel structure of Navpas was used as a template against which the hNav1.1 sequence was modeled (55, 56). Modeling was done according to the protocol established by Bordeli *et al.* (49).

### Molecular docking

The homology model of hNav1.1 and SMILES structure of CBD were imported into Chimera. The structures were energetically minimized, and the lowest energy structure was determined as the most likely molecular interaction.

### Immunocytochemistry

HEK cells stably transfected with the β1-subunit and hNav1.1 α-subunits were incubated for 24 h with 0 (vehicle) and 5 μm CBD. After this incubation period, the cells were fixed in 4% paraformaldehyde for 10 min while being incubated on a shaker. Then cells were incubated in 0.1% Triton X-100 for a further 10 min. The cells were blocked in 10% goat serum for 30–45 min. Following this step, diluted primary anti-hNav1.1 (1:100, mouse monoclonal IgG, commercially available via UC Davis/NIH NeuroMab Facility) antibodies were added. The next day, the cells were incubated in PBS for 10 min. Lastly, secondary Alexa Fluor 546 IgG (1:500) and Hoe33342 (nuclear stain) were added. Stained cells were studied using a Nikon confocal microscope. Images were analyzed using ImageJ.

### Lipid-binding assessment

Made using the TRANSILXL brain absorption kit (ADME Cell) as per the manufacturer's instructions.

### Statistics

A one-factor analysis of variance was used to compare the mean responses. Post hoc tests using the Tukey Kramer adjustment compared the mean responses between channel variants across conditions. A level of significance α = 0.05 was used in all overall post hoc tests, and effects with *p* values less than 0.05 were considered to be statistically significant. All values are reported as means ± S.E. for *n* cells.

## Author contributions

M.-R. G., P. C. R., and S. J. G. conceptualization; M.-R. G. resources; M.-R. G. and S. J. G. data curation; M.-R. G. software; M.-R. G., N. G. S., and S. J. G. formal analysis; M.-R. G. and P. C. R. funding acquisition; M.-R. G. and R. A. D. validation; M.-R. G., P. C. R., and S. J. G. investigation; M.-R. G. visualization; M.-R. G., J. M., and S. J. G. methodology; M.-R. G. writing-original draft; M.-R. G., N. G. S., J. M., R. A. D., P. C. R., and S. J. G. writing-review and editing; P. C. R. and S. J. G. supervision; P. C. R. and S. J. G. project administration.

## Supplementary Material

Supporting Information

## Footnotes

2 The abbreviations used are:

CBD: cannabidiol
Nav: voltage-gated sodium channel
THC: Δ9-tetrahydrocannabinol
Kv: voltage-gated potassium channel
GOF: gain-of-function
LOF: loss-of-function
TTC: tetracaine
SSFI: steady-state fast inactivation.
