# Supplementary material for: Inhibitory effects of cannabidiol on voltage-dependent sodium currents
Source: J Biol Chem. 2018 Sep 14;293(43):16546–58. doi: 10.1074/jbc.RA118.004929 (PMC6204917; doi:10.1074/jbc.RA118.004929)
Supplement: Supporting Information [file supp_RA118.004929_139500_2_supp_201552_pmykhw.docx]

**SUPPORTING INFORMATION**

Inhibitory effects of cannabidiol on voltage-dependent sodium currents

**Mohammad-Reza Ghovanloo ^1,2^, Noah Gregory Shuart ^2^, Janette Mezeyova ^2^, Richard A. Dean ^2^, Peter C. Ruben ^1^ and Samuel J. Goodchild ^2*^**

^1^ Department of Biomedical Physiology and Kinesiology, Simon Fraser University, Burnaby, Canada

^2^ Department of Cellular and Molecular Biology, Xenon Pharmaceuticals, Burnaby, Canada

**FIGURE S1**

**Figure S1 – Molecular Docking of CBD Interaction with F1763 in hNav1.1 & Analysis of Overnight CBD Incubation**

(**A**) Molecular docking prediction of at least one of the CBD binding locations inside the Nav pore. This is a homology model of hNav1.1 based on eukaryotic Navpas. F1763 is highlighted in orange and CBD is indicated in magenta. (**B**) Confocal images of stained HEK cells from the middle confocal plane. The nuclei are stained with Hoe33342 (blue) and hNav1.1 is stained with Alexa Fluor-546 (red) (n = 3). (**C**) Pixel intensity grey value was used as a function of the length of cells (distance) from the middle confocal plane image. For a typical HEK cell with a diameter of about 13 µm, the majority of both vehicle and CBD incubated hNav1.1 channels were localized within the 0 to 3 µm and 11 to 13 µm ranges. These two ranges correspond to the outer cellular edges, indicating that most of the channels seem to be concentrated in the proximity of cell membranes. (**D**) We functionally tested both conditions using whole-cell voltage-clamp. The sodium currents of the CBD-incubated channels were inhibited. In the figure, maximal inactivating current density of HEK cells stably transfected with hNav1.1 is shown (Veh: current density = 280.1 ± 67.5 pA/pF, n = 5; CBD: current density = 18.2 ± 3.5 pA/pF, n = 6). The comparison is between vehicle and 5 µM CBD following overnight incubation. Representative current traces are shown.
